# Aging, Cognitive Efficiency, and Lifelong Learning: Impacts on Simple and Complex Sentence Production During Storytelling

**DOI:** 10.3390/brainsci15101120

**Published:** 2025-10-18

**Authors:** Silvia D’Ortenzio, Francesco Petriglia, Giulia Gasparotto, Sara Andreetta, Marika Gobbo, Andrea Marini

**Affiliations:** 1 Cognitive Neuroscience Laboratory, University of Udine, Via Margreth, 3, 33100 Udine, Italy; silvia.dortenzio@uniud.it (S.D.); francesco.petriglia@unito.it (F.P.); giulia.gasparotto@uniud.it (G.G.); sarandreetta2410@gmail.com (S.A.); gobbomarika@gmail.com (M.G.); 2 Department of Life Sciences, University of Trieste, 34127 Trieste, Italy

**Keywords:** aging, discourse, morphological and syntactic production, cognition, lifelong learning

## Abstract

**Objectives:** This study investigated the effects of healthy aging on sentence production in narrative discourse and examined the role of cognitive abilities and Lifelong Learning (LLL) in mitigating age-related decline. **Methods:** Three hundred and seven Italian-speaking adults (26–89 years) completed a narrative task elicited from five picture stimuli, alongside assessments of verbal working memory, sustained attention, and inhibitory control. Morphological and morphosyntactic measures (morphological errors and omissions of content and function words) and syntactic variables (complete sentences, subordinate clauses, and passive sentences) were analyzed. **Results**: Aging was associated with increased morphological and morphosyntactic errors and reduced syntactic complexity. These effects were non-linear for the % of morphological errors, the % of omission of content words, and the % of complete sentences and were more pronounced after age 70. LLL was negatively associated with morphological and morphosyntactic errors and positively associated with sentence production. Verbal working memory and sustained attention explained additional variance only for omissions of function words, whereas the passive component of verbal working memory only explained additional variance for complete sentence production. **Conclusions:** These findings suggest that aging affects both simple and complex sentence production, with declines related to morphological errors and omissions. LLL appears to buffer against some grammatical declines, suggesting a role for educational engagement in maintaining syntactic abilities. Clinically, assessing complex sentence production and considering LLL may improve diagnosis and intervention for language disorders in older adults.

## 1. Introduction

Recent research has examined the effects of healthy aging on language production [1,2], suggesting that some skills are affected by aging (e.g., speech rate and discourse production skills) while others (e.g., syntactic planning) are apparently preserved [3,4]. The analysis of narrative discourse production is particularly informative, as it highlights the effects of aging on both micro- and macrolinguistic abilities [5,6,7,8,9]. Microlinguistic processes refer to the phonological, lexical, and grammatical aspects of language, whereas macrolinguistic processes concern language use and the integration of meaning across utterances. In this paper we focus on age-related effects on a specific microlinguistic ability (namely, the production of syntactically accurate sentences during narrative discourse production), the impact of lifelong learning on such production across adulthood, and the relation between cognitive and grammatical production abilities.

### 1.1. Age Effects on Morphological and Grammatical Production

Over the past 30 years, several studies have explored how grammatical production abilities are affected by healthy aging. These efforts have yielded mixed results. Some investigations found no age-related differences in grammatical complexity between young and older adults [10,11]. For example, Nippold et al. [11] compared three small groups of healthy adults in their 20s (20 young adults), 40s (20 middle-aged adults), and 60s (20 older adults) using a conversational task about everyday topics and a peer conflict resolution task. They assessed the use of main clauses as well as subordinate clauses, divided into finite (marked for person, tense, and number, e.g., relative, adverbial, and nominal) and non-finite clauses (not marked for these features, e.g., infinitive, participial, and gerundive). No age-related decline was observed in measures of syntactic complexity, suggesting that the ability to produce such sentences remains stable at least through the 60s. In contrast, most investigations have shown that older individuals exhibit declines in grammatical complexity (e.g., [12,13,14]) as they tend to avoid syntactically complex sentences (for an exhaustive definition, see Supplementary Materials) and are more likely to make morphological errors [5,7,15,16,17,18,19]. For example, MacKay & James [19] compared 32 young (aged 18–22) and 32 older adults (aged 67–79) in a task eliciting phonological and morphological speech errors and found that older adults produced significantly more omissions involving inflectional endings (e.g., *mask* instead of *masked*). Focusing on narratives collected from 32 English-speaking adults divided into four age groups (50–59, 60–69, 70–79, and 80–89 years), Kynette and Kemper [18] found that participants in their 70s and 80s produced fewer complex sentences than those aged 50–69. Older participants also struggled with simple syntactic structures, frequently omitting obligatory grammatical morphemes, violating subject–verb agreement, using incorrect tense inflections, and omitting function words (e.g., articles and possessive pronouns). Kemper [17] examined adults’ language production through an imitation task, assessing older (70–89) and younger adults (30–49) on their production of complex sentences varying in grammatical correctness, length, and clause type. Although such controlled tasks involve sentence types that are less frequent in natural speech, they are useful for isolating syntactic processing and memory constraints. Results showed impaired syntactic production in older adults, especially for left-branching sentences, those in which the subordinate clause precedes (e.g., *That the woman has gone away disappointed the husband* or *Looking outside the window distracted the mother*) or interrupts (e.g., *The child*, *whom the girl looks up to*, *is stealing some cookies*) the main clause. In contrast, right-branching sentences (e.g., *The man is angry because his wife has gone away*), where the subordinate clause follows the main clause, were relatively preserved.

In two experiments, Hardy et al. [4] compared syntactic abilities in 56 adults aged 64–80 and 50 younger participants aged 18–25. In the first, they examined the impact of aging on syntactic production speed using a syntactic priming paradigm. Older adults required more time to plan complex sentences, even with syntactic priming, suggesting slower and more error-prone production. However, both age groups benefited from syntactic priming, indicating that syntactic facilitation remains stable with age. In the second experiment, participants’ syntactic planning and lexical retrieval skills were assessed. Older adults were again slower in sentence planning. Although both age groups benefited from a lexical preview (a picture related to the second lexical item), younger participants benefited significantly more, suggesting that aging affects the ability to integrate previewed lexical information during sentence planning. Considering both experiments, Hardy et al. [4] proposed that age-related effects on sentence production may stem from lexical retrieval difficulties rather than a decline in syntactic competence.

A further aspect still requiring systematic investigation concerns the trajectory of age-related decline in grammatical skills. Some evidence suggests that the ability to produce grammatically complete simple sentences declines linearly with age. For example, Marini et al. [20] found that adults in their 70s and 80s produced fewer grammatically well-formed sentences and more morphological errors (mainly inflectional substitutions) than younger participants, following a linear trend. Similar results were reported in larger samples [20]. However, such studies did not assess grammatical skills systematically, leaving open whether different syntactic constructions are affected linearly or non-linearly by aging.

### 1.2. Cognitive Skills Implicated in Morphological and Grammatical Production

The analysis of narrative discourse provides valuable insight into how aging affects language production. Yet, while examining morphological and grammatical skills is essential, it is not sufficient on its own. For example, some studies report increased omissions of inflectional morphemes (e.g., [19]), whereas others suggest that complex morphological processing may remain intact even in individuals with Alzheimer’s disease (e.g., [21]). Thus, morphological alterations may reflect the impact of age-related cognitive constraints rather than a loss of underlying grammatical competence. Similarly, reduced grammatical production may be linked to declines in cognitive efficiency. Indeed, aging influences cognitive abilities supporting morphological and syntactic production, including working memory [16,17,22,23,24,25,26], sustained attention [27], and inhibitory control [28]. These limitations affect the real-time demands of language production, forcing older adults to simplify morphological choices and reduce syntactic complexity. Such adjustments are compensatory strategies that preserve communicative effectiveness despite reduced cognitive efficiency.

Adults over 70 typically show reduced working memory capacity and efficiency [29]. Evidence suggests that working memory supports utterance planning [30] and dependency relations between words in a sentence [31,32]. Hartsuiker and Barkhuysen [33] found that individuals with lower working memory spans had more difficulty producing correct subject–verb agreement. Furthermore, gradual deterioration of working memory has been linked to reduced syntactic complexity in older adults’ utterances. For example, Kemper and Sumner [16] found that 100 younger adults (18–28 years) produced longer and more complex utterances than 100 older adults (63–88 years) and that phonological working memory (as assessed by digit span tasks) was associated with grammatical complexity in both groups, supporting the hypothesis that the gradual reduction in grammatical complexity is related to a reduction in working memory resources. Similar findings were reported by Kynette and Kemper [18] and Kemper [17], who attributed age-related declines in syntactic complexity to reduced working memory efficiency. Producing left-branching sentences likely imposes a heavier memory load and greater attentional demand, as the subordinate clause must be retained in memory while processing the main clause. More recently, comparing language performance of 60 Chinese-speaking adults aged 60–80, Wang and Wang [34] confirmed the age-related decline in the ability to produce complex sentences and highlighted the potential interconnection between such reduced performance and age-related working memory decline.

Sustained attention, the ability to focus cognitive resources over time on specific stimuli [35], is also sensitive to age. It remains relatively stable until the age of 50–69 [36] but declines after 70 [37]. Older adults may compensate by adopting alternative strategies, often at the cost of slower response times [38], which may contribute to the reduced speech rate observed in individuals over 75 [39]. A recent meta-analysis by Vallesi et al. [27] confirmed that aging reduces processing speed in timed tasks and diminishes the ability to keep sustain attention, as reflected in Trail Making Test results [40].

Sustained attention is closely related to executive functions such as inhibitory control [41]. This ability also declines after age 70 [28], potentially compromising morphological and grammatical production. It has been shown to result in less informative and coherent narratives due to the intrusion of irrelevant details–a pattern consistent with the Inhibition Deficit Hypothesis [42], which posits that older adults have reduced ability to suppress irrelevant information and maintain narrative focus. This often manifests as extraneous comments and derailments during storytelling [3,5].

### 1.3. Lifelong Learning Effects on Morphological and Grammatical Production

As mentioned earlier, studies on age-related effects in morphological and syntactic production have yielded mixed results. While most studies report significant effects, others do not. These discrepancies may stem from differences in elicitation tasks or measures. However, education plays a key role in language production. Nippold et al. [11], for example, found no effect of age on grammatical production abilities but noted large individual differences within all three groups. Since these groups did not differ in their years of schooling (a relatively high level of formal education, about three years of college), this suggests that formal education alone may not explain individual variability.

Relying solely on years of schooling provides a limited proxy for educational background, as it captures only early-life formal instruction and not later-life experiences, especially for older individuals. In this regard, the World Health Organization’s *Framework for Active Ageing* [43] recognizes lifelong learning (LLL) as a key factor in promoting participation, health, and safety throughout the ageing process. Broadly speaking, LLL refers to as the ongoing process of acquiring knowledge and skills across the lifespan [44]. Among older adults, it encompasses educational and socially oriented activities aimed at preserving cognitive function, fostering personal development, and supporting social contribution. Such activities may include formal and informal education courses (e.g., language courses, evening classes, art, or music) as well as social activities. Through these forms of learning, older individuals remain active and socially connected [45,46], thereby sustaining their quality of life [47]. In particular, Jenkins et al. [48,49] examined the long-term effects of LLL on active ageing. They found that, in general, higher levels of education are associated with greater well-being; however, in the absence of social–educational interventions, the psychological well-being of older adults tends to decline over time, regardless of their level of education.

Because LLL reflects sustained engagement in cognitively, socially, and professionally stimulating activities that contribute to cognitive reserve [50], incorporating measures of LLL into studies of aging and cognition may provide a more comprehensive and ecologically valid indicator than years of schooling, particularly when investigating language production in adulthood. Previous research has shown that learning among adults aged 50 and above enhances cognitive flexibility [51], self-confidence, life satisfaction, social engagement, and even physical health [52]. Unfortunately, to the best of our knowledge, no studies have yet investigated the relationship between LLL and narrative discourse production in healthy aging.

### 1.4. Research Questions

In summary, prior research shows that aging may impact the production of well-formed sentences, both simple and complex. Older adults produce more morphosyntactic errors (often omitting content and function words) and tend to avoid complex constructions. These difficulties may stem from declines in verbal working memory. However, most studies have been limited by small sample sizes and narrow age ranges (not including the full adult lifespan). Moreover, they rarely considered cognitive abilities beyond working memory. This is a major limitation of the available literature as efficient syntactic production may also depend on other cognitive skills, such as attention and inhibitory control, abilities that decline with age [27,28,53], and can affect word retrieval from memory and the management of interference generated by multiple words during sentence construction [5,54].

This study aims to address these gaps by assessing the effect of healthy aging on sentence production using a narrative task. Specifically, it focuses on two research questions. The first research question (RQ1) examined (a) the impact of aging on the production of both simple (i.e., independent declarative sentences and all clauses that can stand alone as complete sentences, such as main and coordinate clauses) and complex sentences (i.e., those containing a subordinate clause), (b) whether this follows a linear or non-linear trend, and (c) whether Lifelong Learning (LLL) mitigates age-related effects on grammatical production. Based on prior work, we hypothesized that aging would determine an increased production of morphological and morphosyntactic errors (Hypothesis 1a). As a result, it was hypothesized that with increasing age participants would produce fewer complex sentences (Hypothesis 1b). We also hypothesized that such decline would follow a linear trend across syntactic constructions (Hypothesis 1c) and that higher levels of LLL would mitigate this decline (Hypothesis 1d).

The second research question (RQ2) investigated the relationship between verbal working memory, attention, inhibitory control, and the ability to produce simple and complex sentences. Working memory was assessed using the digit recall span task. Sustained attention and inhibitory control were assessed using the Trail Making Test. As complex sentences require the integration of multiple clauses, attentional control, inhibition, and working memory are assumed to play critical roles. We hypothesized that these cognitive skills that in previous studies have been found to decline with age would be related to grammatical production (Hypothesis 2).

## 2. Materials and Methods

### 2.1. Participants

Three hundred and seven Italian-speaking healthy adults aged 26 to 89 years were recruited for this study. Their demographic and general characteristics are reported in Table 1.

Participants were recruited through lifelong learning institutes, local social or sport clubs, voluntary and charitable associations, and personal contacts using informative flyers and advertisements on social networks. Inclusion criteria were the absence of neurological or neuropsychiatric disorders, performance above the cutoff on the Italian version of the Montreal Cognitive Assessment (MoCA) [55], and the naming subtest of the Italian version of the Aachen Aphasia Test (AAT) [56].

**Table 1:** General information about the participants in the study.

	26–29	30–39	40–49	50–59	60–69	70–79	80–89
Age	27.43 (1.12)	34.15 (2.59)	44.87 (2.83)	54.86 (2.68)	62.97 (2.74)	74.52 (3.18)	83.04 (2.56)
Education	15.95 (2.73)	16.67 (3.45)	14.23 (3.98)	14.44 (3.35)	11.60 (4.49)	10.90 (4.04)	7.48 (3.47)
LLL	103.16 (11.73)	108.92 (11.88)	108.68 (17.70)	113.03 (13.14)	107.54 (18.43)	108.17 (14.50)	101.61 (13.77)
Sex	F = 52%	F = 60%	F = 58%	F = 73%	F = 54%	F = 52%	F = 68%
MoCA	27.76 (1.70)	28.42 (1.51)	28.19 (1.78)	27.33 (1.63)	27.46 (1.64)	26.45 (2.10)	27.88 (2.22)
Token	4.97 (0.12)	5.00 (0.00)	5.00 (0.00)	4.98 (0.10)	4.89 (0.63)	4.90 (0.21)	4.95 (0.18)
Naming_AAT	118.21 (1.93)	119.00 (1.54)	118.83 (2.40)	118.20 (2.12)	118.09 (2.11)	116.17 (2.79)	115.96 (4.13)
Words (total)	535.10 (217.84)	566.73 (222.72)	468.13 (214.16)	485.90 (186.36)	442.54 (212.37)	507.84 (213.29)	334.40 (152.73)
Words (mean)	103.28 (41.43)	110.02 (42.74)	92.05 (42.87)	94.67 (35.40)	84.80 (40.20)	98.16 (40.69)	64.87 (29.92)

Data are presented as means, with standard deviations in parentheses, for each age group. For sex, the percentage of female participants is reported. Legend: MoCA = Montreal Cognitive Assessment [55]; LLL = Lifelong Learning, as assessed using the *Education* section of the Cognitive Reserve Index Questionnaire [57]; AAT = Aachen Aphasia Test [56]. *Words (total)* and *Words (mean)* refer to the total number of words and the mean number of words produced by participants across the five picture stimuli in the narrative production task.

As can be seen in Table 1, the participants in their 70s and 80s showed lower levels of formal education (i.e., years of schooling) compared to younger groups. This is not an uncommon finding in studies involving Italian speakers. This is because access to schooling was more limited in the first half of the 20th century, particularly in rural areas. For this reason, in this study participants’ Lifelong Learning (LLL) levels were used as a measure of their level of education. Specifically, LLL was determined using the *Education* section of the Cognitive Reserve Index Questionnaire (CRIq) [57], which considers not only years of formal education at school but also additional training courses lasting at least six months completed during adulthood. All groups produced stories that exceeded the 300-word threshold, which is considered adequate to ensure high test–retest stability even in clinical settings [58].

This study was not preregistered. The Ethical Committee of the University of Udine approved the research (protocol CGPER-2024-02-27-01). All participants provided informed consent prior to participation.

### 2.2. Assessment of Discourse Production

Five picture stimuli were used to collect narratives and assess participants’ narrative abilities: two single-picture scenes, the *Picnic* from the Western Aphasia Battery [59] and the *Cookie Theft* by Goodglass and Kaplan [60], and three cartoon picture sequences, the *Flowerpot* by Huber and Gleber [61], the *Quarrel* by Nicholas and Brookshire [62], and the *Nest Story* by Paradis and Libben [63]. The use of five stories ensured that participants’ production exceeded the 300-word threshold considered necessary for reliable analyses [58].

To avoid referent sharing with the examiner, each stimulus was randomized and presented on a laptop with the screen facing the participant. Before the assessment, participants were shown the following message on the screen: “You will see stories in the form of images. The images are randomly selected by the program, so I don’t know them. As soon as the image appears, describe it to me. There is no time limit and no right or wrong way to describe them. You can speak as much or as little as you like. However, avoid using words like ‘here,’ ‘this,’ etc., and be as clear as possible.” The narratives were audio-recorded.

Speech samples were transcribed using a semi-automatic procedure developed by one of the authors (F.P.), consisting of an automated Python workflow hosted on Google Colab to expedite transcription and analysis. All analyses were conducted between August and November 2024 with Python (v3.12). Automatic Speech Recognition (ASR) was performed using Whisper (version 20231117) [64]. The Whisper large-v3-model [64] was used to transcribe the participants’ narrative recordings into raw text, excluding repetitions, rephrasing, and false starts. The Spacy Python library (v3.7.4) [65] processed the transcriptions by adding lemmatization and Part-Of-Speech (POS) tagging, while pandas (v2.2.2) was used for data management and analysis. Each automatized transcription was controlled by a human annotator, who restored omitted elements, corrected tagging errors, and resolved ambiguities in word classification.

To evaluate the reliability of the Python pipeline, we validated it on a subsample of 103 transcripts. Each automatically generated text was compared against its manually validated version using the Levenshtein distance [66], which measures the minimum number of insertions, deletions, or substitutions required to convert one sequence into another. Analyses were performed at the word level. On average, 25 word-level operations were required, involving 18.51% of the total words. At the label level, the average number of modifications was higher, affecting 33.50% of total labels (mean of 53 label-level modifications). We further performed correlation analyses to identify which POS categories showed the strongest agreement between human and automatic annotations. The strongest correlations were observed for core lexical categories such as determiners (ρ = 0.954), verbs (ρ = 0.958), and nouns (ρ = 0.932). In contrast, some specific categories from the multilevel discourse analysis framework (e.g., tangential words or content word omissions) were never assigned by the pipeline. For transparency and reproducibility, all code and analysis scripts used to generate transcripts from audio are openly available in a dedicated GitHub repository (https://github.com/FraPet/SMOOTH-asr_nlp_pipeline, accessed on 11 August 2025).

The final transcripts included phonological fillers, pauses, false starts, phonological errors, and neologisms. Self-corrections and restarts were retained in both transcriptions and analyses. Utterances were segmented based on the four criteria described in Marini et al. [67]: acoustic, semantic, grammatical, and phonological. According to the acoustic criterion, an utterance is considered interrupted when a long pause occurs (e.g., “There is a … (10 s)/man.”). The semantic criterion defines an utterance as complete when the proposition is conceptually whole and uninterrupted (e.g., “There is a man who walks down the street.”). The grammatical criterion establishes segmentation when a sentence is grammatically complete and free of pauses or propositional violations, possibly including subordinate clauses (often overlapping with the semantic criterion, as in “There is a man who walks down the street when a flowerpot falls on his head.”). Finally, the phonological criterion considers an utterance interrupted when a word itself is broken off, as in false starts (e.g., “There is a ma-/ma-/man walking down the street”).

Following Marini et al. [67], morphosyntactic errors were assessed in terms of percentages of morphological errors and omissions of content and function words. Sentence production skills were assessed by calculating the percentage of complete sentences (i.e., grammatically well-formed sentences; those with omissions or morphological errors were excluded). For this study, one of the authors (S.D.) devised additional measures, including the percentage of subordinate clauses, adverbial clauses, complement clauses, relative clauses, and passive sentences.

Considering morphosyntactic measures, the percentage of morphological errors was calculated by dividing the number of such errors by the total number of utterances and then multiplying by 100. The percentage of the omission of content words (i.e., nouns, verbs, adverbs, or adjectives) was determined dividing the number of omissions of such words by the total number of utterances and multiplying the result by 100. Similarly, the percentage of the omissions of function words (e.g., articles, prepositions, etc.) was calculated dividing the number of such omissions by the total number of utterances and then multiplying by 100.

Considering syntactic measures, the percentage of complete sentences (i.e., all grammatically well-formed sentences, excluding those with omissions or morphological errors) was calculated by dividing the number of utterances that were scored as complete sentences by the total number of utterances and then multiplying by 100. The percentage of subordinate clauses was calculated by dividing the number of subordinate clauses by the total number of utterances and then multiplying by 100. The percentage of complement clauses (i.e., subordinate clauses that completed the verb argument structure) was calculated by dividing the total number of clauses that were scored as grammatical by the total number of utterances and then multiplying by 100. The percentage of adverbial clauses (i.e., subordinate clauses serving as adjuncts to the main clause) was calculated by dividing the total number of grammatical clauses by the total number of utterances and then multiplying by 100. The percentage of relative clauses (i.e., subordinate clauses modifying a nominal element) was determined by dividing the total number of clauses that were scored as correct by the total number of utterances and then multiplying by 100. Finally, the percentage of passive sentences was calculated by dividing the number of such sentences that were scored correct by the total number of utterances and then multiplying by 100.

### 2.3. Cognitive Assessment

The cognitive assessment focused on verbal working memory, sustained attention, and inhibitory control abilities.

Verbal working memory was assessed using the Forward and Backward Digit Recall tests [68]. Forward Digit Recall evaluates the passive component of verbal working memory. Participants were asked to repeat sequences of digits spoken by the examiner. The test began with sequences of three digits and gradually increased to nine. Each sequence length was presented twice (e.g., 7-9-2 and 6-1-8). Participants received one point for each correctly repeated sequence, and their span corresponded to the longest sequence correctly repeated at least once. The Backward Digit Recall Task assessed the active component of verbal working memory, namely the ability to manipulate verbal information [69]. Participants were required to repeat digits sequences in reverse order. As in the forward version, each sequences length was presented twice (e.g., 8-4-9 and 1-5-3), with the number of digits increasing from three to eight. Participants received one point for each correctly repeated sequence, and the span was defined as the longest sequence correctly repeated at least once.

Sustained attention and inhibitory control were assessed using the Trail Making Test (TMT) [70]. The TMT comprises two parts, each consisting of 25 circles distributed across a sheet of paper. In Part A, each circle contains a number (1–25) and participants are instructed to connect the circles in ascending numerical order as quickly as possible. In Part B, the circles contain both numbers (1–13) and letters (A–N); participants must connect them in alternating sequence (e.g., 1-A-2-B, etc.). The time required to complete Part A is usually considered a measure of sustained attention, whereas the time required to complete Part B is interpreted as reflecting both sustained attention and inhibitory control. The B/A ratio can be considered as an indirect measure of inhibitory control [71].

### 2.4. Statistical Analyses

All analyses were conducted in JASP (version 0.19.3). Regarding the first research question (RQ1) to examine whether age predicted the outcome variables, simple linear regression analyses were first performed with Age entered as the sole predictor. To examine whether the effect of Age was linear or non-linear, these models were extended by including a quadratic term (Age^2^). Improvement in model fit was evaluated using significance tests. When the quadratic term was not significant, the linear model was retained for interpretation. Finally, to test whether Lifelong Learning (LLL) explained additional variance in the grammatical variables beyond Age, multiple regression models were fitted including both Age and LLL as predictors. For all analyses, model fit indices (R^2^) and regression coefficients with associated *p*-values were reported.

To address the second research question (RQ2: whether cognitive measures explained additional variance in the linguistic outcomes beyond demographic factors), a series of hierarchical multiple regression analyses were performed. In the first block, Age and LLL were entered as predictors to account for demographic effects. In the second block, the cognitive variables (i.e., Forward digit recall, Backward digit recall, TMT_A, and TMT_B/A) were entered. All predictors were standardized prior to analyses by calculating z-scores. This allowed assessment of the incremental variance explained by the cognitive measures after controlling for Age and LLL. Model fit was evaluated at each step using R^2^, and the change in explained variance (ΔR^2^) between blocks was tested for statistical significance. For each predictor, standardized coefficient (β) and associated *t*-tests were reported. Because multiple cognitive predictors were tested within the same model, *p*-values for these effects were adjusted using the Holm–Bonferroni correction to control for the familywise error rate.

### 2.5. Sample Size Estimation

For the multiple linear regression analyses we followed the recommendations by Stevens [72], who suggests that 15 subjects per predictor are needed for a reliable equation. Even using the more stringent methodology by Tabachnick and Fidell [73], who suggest using the formula N > 50 + 8 m (where m = number of independent variables), the minimum number of participants should be 98.

To further explore the actual power of the study, a post hoc power analysis using G*Power 3.1 was conducted for the overall multiple linear regression model that included all six independent variables entered simultaneously for % Complete sentences (R^2^ = 0.204), sample size (N = 307), and significance level (α = 0.05). The corresponding Cohen’s *f*^2^ was 0.256. Using this effect size, the achieved statistical power (1-β) for the omnibus test of the full model was approximately 1.00. This confirms that the sample size provided excellent power, ensuring sufficient sensitivity for the regression analyses.

## 3. Results

### 3.1. Age- and LLL-Related Effects on Morphological and Morphosyntactic Production Abilities

Results concerning the participants’ morphological and morphosyntactic abilities are shown in Table 2.

**Figure 1:**
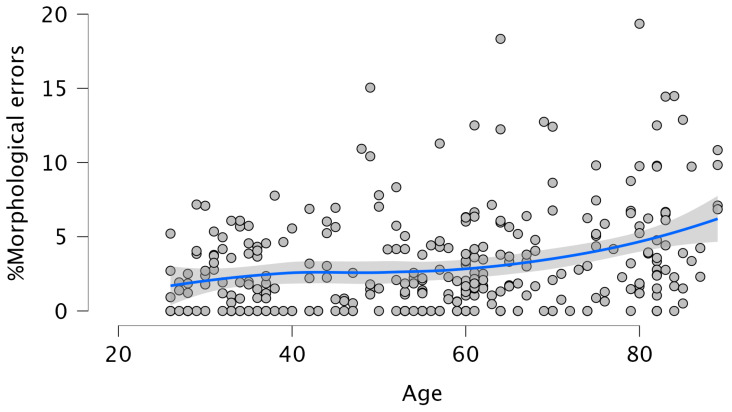
The non-linear effect of Age on % Morphological errors. The blue line represents the Smooth regression line. The grey area shows 95% confidence intervals.

Age was positively associated with the percentage of morphological errors (β = 0.280, *p* = 0.001), accounting for 7.8% of the variance (F (1, 306) = 25.959, *p* = 0.001; R^2^ = 0.078). However, this effect was non-linear (see Figure 1): when a quadratic term for Age was included, the quadratic effect was significant (β = −0.881, *p* = 0.001) and explained an additional 14.5% of the variance (ΔR^2^ = 0.145), indicating a curvilinear association between Age and morphological errors. Beyond the effect of Age, Lifelong Learning (LLL) was negatively associated with morphological errors (β = −0.215, *p* = 0.001), explaining 12.5% of the variance in the outcome (F (2, 304) = 21.668, *p* = 0.001; R^2^ = 0.125). Importantly, the effect of Age remained significant even after accounting for LLL (β = 0.263, *p* = 0.001).

**Figure 2:**
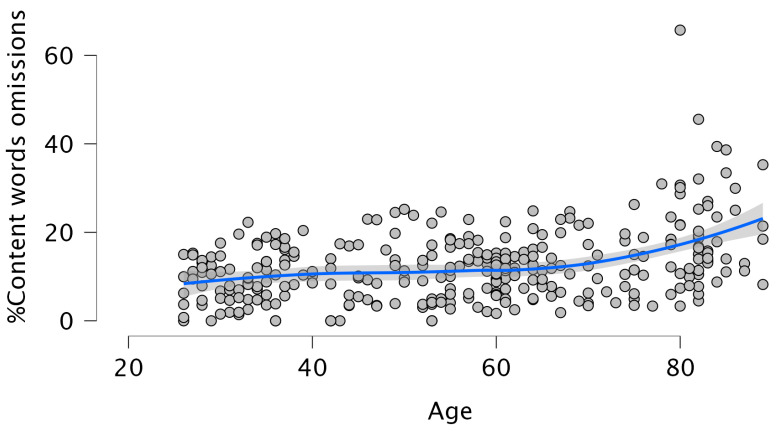
The non-linear effect of Age on % Omission of content words. The blue line represents the Smooth regression line. The grey area shows 95% confidence intervals.

Age was positively associated with the percentage of omissions of content words (β = 0.361, *p* = 0.001), accounting for 13% of the variance (F (1, 306) = 45.604, *p* = 0.001; R^2^ = 0.130). This effect was non-linear (see Figure 2): the quadratic term for Age was significant (β = 1.077, *p* = 0.002) and explained an additional 14.6% of the variance (ΔR^2^ = 0.146), indicating a curvilinear association between Age and the omission of content words. Beyond the effect of Age, Lifelong Learning (LLL) was negatively associated with content word omissions (β = −0.116, *p* = 0.031), accounting for 14.3% of the variance (F (2, 304) = 25.114, *p* = 0.001; R^2^ = 0.143). The effect of Age remained significant in this model (β = 0.349, *p* = 0.001).

For omissions of function words, Age was also positively associated (β = 0.244, *p* = 0.001), explaining 5.9% of the variance (F (1, 306) = 19.285, *p* = 0.001; R^2^ = 0.059). Unlike content words, this effect was linear: the quadratic term was not significant (β = −0.381, *p* = 0.303). When LLL was entered alongside Age, it was not a significant predictor of function word omissions, whereas Age continued to show a significant positive association.

### 3.2. Age- and LLL-Related Effects on Syntactic Production Abilities

Results concerning the participants’ morphological and morphosyntactic abilities are shown in Table 3.

**Figure 3:**
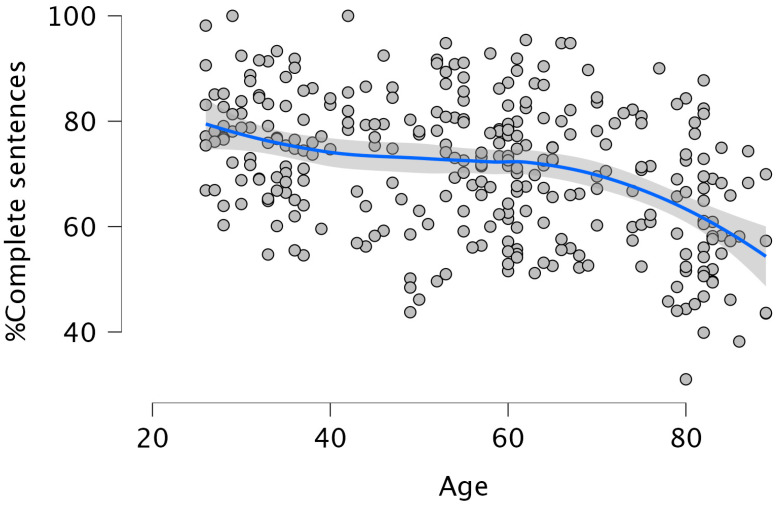
The non-linear effect of Age on % Complete sentences. The blue line represents the Smooth regression line. The grey area shows 95% confidence intervals.

Age was negatively associated with the percentage of complete sentences (β = −0.387, *p* = 0.001), accounting for 15% of the variance (F (1, 306) = 53.690, *p* = 0.001; R^2^ = 0.150). This effect was non-linear (see Figure 3): the quadratic term was significant (β = −0.881, *p* = 0.013), explaining an additional 14.5% of the variance (ΔR^2^ = 0.145), suggesting a curvilinear association between Age and complete sentence production. Beyond Age, Lifelong Learning (LLL) was positively associated with complete sentences (β = 0.178, *p* = 0.001), explaining 18.1% of the variance (F (2, 304) = 33.342, *p* = 0.001; R^2^ = 0.181). The effect of Age remained significant in this model (β = −0.370, *p* = 0.001).

**Figure 4:**
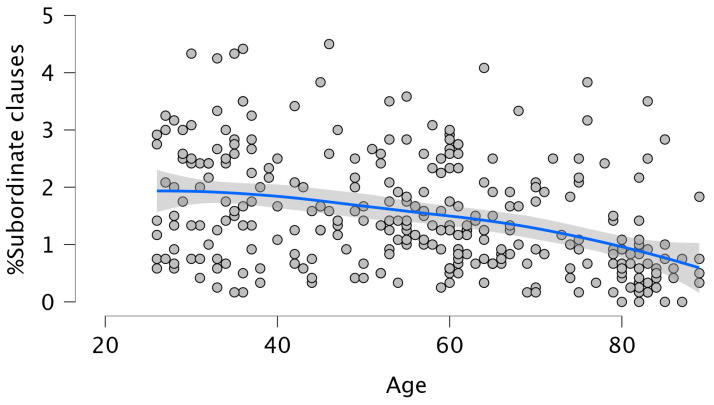
The linear effect of Age on % Subordinate clauses. The blue line represents the Smooth regression line. The grey area shows 95% confidence intervals.

Similarly, Age was negatively associated with the percentage of subordinate clauses (β = −0.369, *p* = 0.001), accounting for 13.6% of the variance (F (1, 306) = 48.203, *p* = 0.001; R^2^ = 0.136). This effect was linear (see Figure 4), as the quadratic term was not significant (β = 0.159, *p* = 0.653). LLL was positively associated with subordinate clause use (β = 0.224, *p* = 0.001), explaining 18.7% of the variance (F (2, 304) = 34.722, *p* = 0.001; R^2^ = 0.187), while the effect of Age remained significant (β = −0.350, *p* = 0.001).

For relative clauses, Age showed a negative association (β = −0.280, *p* = 0.001), explaining 7.8% of the variance (F (1, 306) = 25.861, *p* = 0.001; R^2^ = 0.078). This effect was linear, as the quadratic term was not significant (β = −0.191, *p* = 0.602). After controlling for Age, LLL did not significantly predict relative clause use (β = 0.098, *p* = 0.078), although the effect of Age remained significant (β = −0.277, *p* = 0.001). The model explained 9.1% of the variance (F (2, 304) = 15.100, *p* = 0.001; R^2^ = 0.091).

Age was also negatively associated with complement clause use (β = −0.289, *p* = 0.001), accounting for 8.4% of the variance (F (1, 306) = 27.801, *p* = 0.001; R^2^ = 0.084). This effect was linear, as the quadratic term was not significant (β = −0.094, *p* = 0.797). LLL was positively associated with complement clauses (β = 0.175, *p* = 0.001), explaining 11.9% of the variance (F (2, 304) = 20.305, *p* = 0.001; R^2^ = 0.119), while Age remained significant (β = −0.281, *p* = 0.001).

For adverbial clauses, Age showed a negative association (β = −0.363, *p* = 0.001), accounting for 13.2% of the variance (F (1, 306) = 46.388, *p* = 0.001; R^2^ = 0.132). This effect was linear, as the quadratic term was not significant (β = 0.517, *p* = 0.147). LLL was positively associated with adverbial clauses (β = 0.224, *p* = 0.001), explaining 18.2% of the variance (F (2, 304) = 33.661, *p* = 0.001; R^2^ = 0.182), and Age continued to be significant (β = −0.344, *p* = 0.001).

Finally, Age was negatively associated with the percentage of passive clauses (β = −0.175, *p* = 0.002), accounting for 3% of the variance (F (1, 306) = 25.861, *p* = 0.001; R^2^ = 0.030). This effect was linear, as the quadratic term was not significant (β = −0.551, *p* = 0.143). LLL was positively associated with passive clause use (β = 0.212, *p* = 0.001), explaining 7.4% of the variance (F (2, 304) = 12.146, *p* = 0.001; R^2^ = 0.074), while the effect of Age remained significant (β = −0.154, *p* = 0.006).

### 3.3. Cognitive Effects on Morphological and Morphosyntactic Production

Results concerning the participants’ performance on cognitive tasks are shown in Table 4.

Age and education together accounted for 13% of the variance in morphological errors (F (3, 304) = 15.005, *p* = 0.001; R^2^ = 0.130). When cognitive measures were added, the full model explained 15% of the variance (F (6, 304) = 8.463, *p* = 0.001; R^2^ = 0.146), but the improvement over age and education alone was not significant (ΔF (3, 298) = 1.802, *p* = 0.147; ΔR^2^ = 0.015). None of the cognitive measures significantly predicted morphological errors beyond age and education.

For omissions of content words, age and education explained 14.5% of the variance (F (3, 304) = 17.019, *p* = 0.001; R^2^ = 0.145). The inclusion of cognitive variables raised the explained variance only slightly to 15% (F (6, 304) = 8.793, *p* = 0.001; R^2^ = 0.150) and this increase was not significant (ΔF (3, 298) = 0.629, *p* = 0.597; ΔR^2^ = 0.005). None of the cognitive measures emerged as significant predictors.

In contrast, for omissions of function words, age and education accounted for 9.2% of the variance (F (4, 304) = 7.629, *p* = 0.001; R^2^ = 0.092). Adding cognitive measures significantly improved prediction, increasing explained variance to 11.6% (F (6, 304) = 6.498, *p* = 0.001; R^2^ = 0.116; ΔF (2, 298) = 0.629, *p* = 0.021; ΔR^2^ = 0.023). In the final model, Forward Digit Recall (β = −0.175, *p* = 0.005) and TMT_A (β = 0.173, *p* = 0.014) were significant predictors, indicating that poorer short-term memory and reduced processing speed were associated with more omissions of function words.

### 3.4. Cognitive Effects on Syntactic Production

Age and education together accounted for 18.3% of the variance in complete sentence production (F (3, 304) = 22.425, *p* = 0.001; R^2^ = 0.183). Adding cognitive measures significantly improved prediction (ΔF (3, 298) = 2.649, *p* = 0.049; ΔR^2^ = 0.021), increasing the explained variance to 20.4% (F (6, 304) = 12.721, *p* = 0.001; R^2^ = 0.204). In the final model, Forward Digit Recall emerged as a significant positive predictor (β = 0.156, *p* = 0.009), indicating that better short-term memory was associated with producing more complete sentences.

For subordinate clauses, age and education explained 18.9% of the variance (F (3, 304) = 23.459, *p* = 0.001; R^2^ = 0.189). The full model including cognitive measures explained 20.5% of the variance (F (6, 304) = 12.841, *p* = 0.001; R^2^ = 0.205), but this improvement was not significant (ΔF (3, 298) = 1.992, *p* = 0.115; ΔR^2^ = 0.016), and no cognitive variable was a unique predictor.

Adverbial clause use showed a similar pattern: age and education explained 18.5% of the variance (F (3, 304) = 22.838, *p* = 0.001; R^2^ = 0.185). With cognitive measures added, explained variance rose slightly to 20.3% (F (6, 304) = 12.684, *p* = 0.001; R^2^ = 0.203), but the improvement was not significant (ΔF (3, 298) = 2.245, *p* = 0.083; ΔR^2^ = 0.018), and none of the cognitive variables significantly predicted adverbial clause production.

For complement clauses, age and education explained 12.2% of the variance (F (3, 304) = 13.962, *p* = 0.001; R^2^ = 0.122). Adding cognitive measures raised the explained variance to 13.6% (F (6, 304) = 7.807, *p* = 0.001; R^2^ = 0.136), but the increase was not significant (ΔF (3, 298) = 1.572, *p* = 0.196; ΔR^2^ = 0.014), and no cognitive variable was a significant predictor.

Relative clauses showed a different pattern: age and education explained 9.1% of the variance (F (2, 304) = 15.100, *p* = 0.001; R^2^ = 0.091). Adding cognitive measures significantly improved prediction (ΔF (4, 298) = 2.690, *p* = 0.031; ΔR^2^ = 0.032), raising explained variance to 12.3% (F (6, 304) = 6.939, *p* = 0.001; R^2^ = 0.123). However, none of the individual cognitive variables reached significance in the final model.

Finally, for passive sentences, age and education explained 7.5% of the variance (F (3, 304) = 8.150, *p* = 0.001; R^2^ = 0.075). With cognitive measures included, explained variance increased slightly to 8.4% (F (6, 304) = 4.561, *p* = 0.001; R^2^ = 0.084), but this change was not significant (ΔF (3, 298) = 0.974, *p* = 0.405; ΔR^2^ = 0.009). None of the cognitive variables uniquely predicted passive sentence use.

## 4. Discussion

This study examined how healthy aging and lifelong learning (LLL) influence adults’ morphological and syntactic production abilities and how these language skills relate to cognitive resources such as attention, inhibitory control, and working memory. Specifically, we investigated a) whether aging affects the production of simple and complex sentences, b) whether these effects follow a linear or non-linear trend, and c) whether engagement in LLL mitigates age-related declines in grammatical production. A second goal was to assess how cognitive abilities contribute to morphological and syntactic performance.

Overall, the results partially supported our hypotheses. Aging was consistently associated with increased morphological and morphosyntactic errors and reduced syntactic complexity. This confirms that grammatical production declines with age (Hypotheses 1a and 1b). However, these effects were often non-linear, with performance showing a curvilinear trajectory (partially confirming Hypothesis 1c). Specifically, the effect was non-linear for the % of morphological errors, the % of omission of content words, and the % of complete sentences. Looking at Figure 1, Figure 2 and Figure 3, it can be observed that these declines are more pronounced at later ages rather than proceeding gradually. This is consistent with previous studies [3,4,5,17,18,34]. For example, in [5] significant declines in several aspects of discourse production were reported after the age of 75. Regarding the production of complex sentences, when all subordinate clauses were considered together, older adults produced fewer such structures than younger participants, following a linear decline with age. To the best of our knowledge, this has not been highlighted in previous investigations, possibly due to small sample sizes or a failure to examine the entire adult lifespan. Interestingly, the analyses revealed that the decrease in the production of the different types of subordinate clauses was always linear, with a marked decline between 70 and 80 years. We also found a significant age-related decrease in the production of passive sentences. However, the production of such sentences was so small that this finding should be treated with caution. An inspection of the correlations between omissions of content words, omissions and function words and the production of the different types of subordinate clauses revealed the absence of any relation between omissions of content words and the production of such sentences. In contrast, the omissions of function words showed significant negative correlations with % Relative clauses (r = −0.174; *p* < 0.002), % Complement clauses (r = −0.197; *p* < 0.001), % Adverbial clauses (r = −0.167; *p* < 0.003), and % Passive sentences (r = −0.131; *p* < 0.022), a pattern also reported by Kynette and Kemper [18].

These findings suggest that older adults tend to produce fewer subordinate clauses and passive sentences because they omit the words necessary to link the subordinate to the main clause resulting in incomplete and ungrammatical sentences. Additionally, subordinate clauses present syntactic challenges such as embedding and dependency, making them more error-prone. Embedding involves placing one clause within another, which can disrupt word order, require clause extraposition, or introduce backwards pronominal anaphora. Dependency refers to the fact that subordinate clauses cannot stand alone; they require specific conjunctions or dependent verb forms [74]. Passive sentences also involve complex syntactic operations, such as reassignment of syntactic functions through constituent movement. Their complexity may explain why they are impaired not only in older adults but also in other populations such as university students with dyslexia [75] and persons with aphasia [76,77].

Importantly, LLL exerted a protective influence confirming Hypothesis 1d. Previous studies showed that LLL has positive effects as it enhances confidence [78,79,80,81], interpersonal skills [82], employment opportunities [82], and cognitive abilities [83,84,85], with downstream benefits for communication skills. In our study, even after controlling for age, LLL was negatively associated with morphological errors and with content word omissions and positively associated with complete sentences, subordinate clause use, complement clauses, adverbial clauses, and passive clauses. Overall, these findings align with the view that sustained intellectual engagement may buffer against age-related declines in those abilities that support sentence production. In other words, individuals who engage in continuous education tend to produce more syntactically complex language. Indeed, engaging in learning activities in later life plays an important role in developing and maintaining a set of psychological (e.g., “self-esteem and self-efficacy”, “sense of purpose”, and “social integration”), cognitive, and communicative resources that contribute to promoting active ageing and mental health [51,52]. In this regard, given the global trend towards population ageing, it is increasingly essential to examine the impact of learning in later life, which plays a crucial role in supporting the autonomy and overall quality of life of older people [47,48,49]. The result of a link between LLL and sentence production ability supports this view. Additionally, it shows that an active learning engagement throughout life exerts an influence on linguistic function in old age.

From a clinical point of view, this is an interesting finding as it highlights that language assessment in adults with acquired brain disorders should consider not only age and years of schooling, but also other critical factors that may affect patients’ performance on language tasks. An accurate assessment should therefore include also the administration of questionnaires (such as the CRIq used in this study) to gather information about the patient’s LLL history. Nonetheless, it is noteworthy that in our study LLL did not affect the omissions of function words. This is likely because such function words are characterized by extremely high frequency in language and are therefore usually produced using more automated, procedural, processes, rather than declarative memory.

Regarding RQ2, when cognitive predictors were considered, the passive component of verbal working memory and sustained attention (as measured by Forward Digit Recall and TMT_A, respectively) explained additional variance only for omissions of function words, whereas the passive component of verbal working memory only explained additional variance for complete sentence production. Working memory is crucial for maintaining syntactic dependencies [31,32] and its limitations can hinder processing of sentences with multiple or long dependencies, which is a challenge often faced by older adults. Overall, this pattern suggests that while domain-general cognitive resources contribute to certain aspects of grammatical production, age and experiential factors such as LLL may play a broader and more consistent role in maintaining morphological and syntactic accuracy (partial confirmation of Hypothesis 2).

The findings of this study are also interesting for their clinical application. Identifying difficulties in producing complex sentences can provide diagnostic value, particularly in persons with mild language deficits or difficulties that are not easily detectable through standard tests. Consider, for example, persons with post-stroke aphasia. These patients might obtain scores within normal range on traditional standardized aphasia batteries (e.g., AAT) but still experience residual deficits or report discrepancies between their pre- and post-event linguistic performance. The assessment of their ability to produce complex syntactic sentences may be helpful to identify the presence of a language alteration and provide diagnostic material to enroll them in Speech-Language Therapy (SLT) programs. Similarly, the assessment of grammatical complexity could be beneficial for persons with primary progressive aphasia (PPA), a neurodegenerative disease starting mostly with a difficulty in the language domain. According to Gorno Tempini’s classification [86], the non-fluent variant of PPA is characterized, among the other things, by disorders in grammatical processing. An accurate assessment of the ability to produce complex sentences could facilitate the early detection of such disorders, allowing for timely recourse to SLT interventions aimed at preserving linguistic functions and mitigating the progression of linguistic deterioration. Overall, this study provides new relevant information which possibly guides clinicians in delineating the actual levels of grammatical functioning in adults.

This study has some limitations. First, the narrative task based on five picture stimuli could not elicit the full range of complex sentence structures available to a speaker. Other elicitation methods (such as discussing interpersonal problems [87] or explaining rules and strategies for games or sports [11]) may yield more syntactically complex productions. Second, our sample included only healthy participants, limiting the generalizability of this study to populations with brain lesions, psychiatric conditions, or neurodegenerative diseases. Third, as ours is a study with a cross-sectional design, we could not directly interpret LLL as a protective factor. Future studies should employ longitudinal designs to directly explore the relationship between LLL and syntactic production. Finally, the B/A ratio used in this study to assess inhibitory control is an indirect measure of this complex ability. Future investigations should assess the effect of inhibitory control on morphological and syntactic production using more adequate and direct tasks such as Flanker or Stroop tests.

Despite these limitations, this study advances our understanding of complex sentence production in healthy aging and its relation to cognition. It also highlights the effects of LLL in preserving grammatical abilities. These findings also have clinical implications: including an analysis of complex sentence production in the assessment of language disorders may inform more targeted interventions to improve quality of life not only for patients with language disorders but also for older adults more generally.

## 5. Conclusions

This study provides new insights into how healthy aging impacts grammatical production in narrative contexts. Moreover, it is suggested that Lifelong Learning may help mitigate the effect of age on communicative competence. These findings offer valuable implications for both research and clinical practice and for the development of interventions addressed to support communication in older adults, enhancing their quality of life and social engagement.

## Figures and Tables

**Table 2:** Morphological and morphosyntactic production of the participants.

	26–29	30–39	40–49	50–59	60–69	70–79	80–89	Age Effect	LLL Effect	Cognitive Effect
%Morphological errors	1.63 (2.01)	2.36 (2.17)	3.00 (3.84)	2.22 (2.53)	3.33 (3.39)	3.84 (3.40)	5.09 (4.46)	Non- linear	Yes	None
% Omission of content words	9.04 (5.14)	10.23 (5.99)	10.30 (6.95)	11.35 (6.70)	11.79 (5.63)	12.78 (7.37)	19.75 (11.88)	Non- linear	Yes	None
% Omission of function words	0.32 (0.69)	0.55 (1.0)	0.79 (1.69)	0.79 (1.53)	0.95 (1.60)	1.41 (2.09)	1.92 (3.04)	Linear	No	Forward digit recall TMT_A

Data are presented as means with standard deviations in parentheses for each age group. The table also shows if the age effect was linear or non-linear and whether the effect of LLL was significant. Legend: LLL = Lifelong Learning; TMT_A = Trail Making Test, Part A.

**Table 3:** Syntactic production abilities of the participants.

	26–29	30–39	40–49	50–59	60–69	70–79	80–89	Age Effect	LLL Effect	Cognitive Effect
% Complete sentences	78.74 (10.02)	75.20 (10.36)	72.86 (13.77)	74.14 (11.91)	70.81 (12.48)	68.77 (12.11)	60.27 (13.04)	Non- linear	Yes	Forward digit recall
% Subordinates clauses	1.84 (0.96)	1.99 (1.14)	1.69 (1.02)	1.60 (0.79)	1.40 (0.84)	1.36 (0.94)	0.76 (0.71)	Linear	Yes	None
% Relative clauses	2.73 (1.46)	2.52 (1.28)	2.05 (1.36)	2.13 (1.56)	1.87 (1.46)	2.34 (1.74)	1.09 (0.88)	Linear	No	None
% Complement clauses	2.26 (1.24)	2.03 (1.06)	2.00 (1.11)	1.76 (0.92)	1.55 (0.94)	1.77 (1.09)	1.11 (0.88)	Linear	Yes	None
% Adverbial clauses	1.94 (1.02)	2.14 (1.21)	1.74 (1.04)	1.69 (0.84)	1.51 (0.91)	1.47 (1.02)	0.82 (0.75)	Linear	Yes	None
% Passive sentences	0.15 (0.22)	0.14 (0.19)	0.12 (0.20)	0.14 (0.19)	0.12 (0.17)	0.07 (0.15)	0.05 (0.11)	Linear	Yes	None

Data are presented as means with standard deviations in parentheses for each age group. The table also shows if the age effect was linear or non-linear and whether the effect of LLL was significant.

**Table 4:** Performance on cognitive tasks.

	26–29	30–39	40–49	50–59	60–69	70–79	80–89
Forward digit recall_span	6.76 (1.04)	6.27 (1.11)	6.32 (1.05)	6.06 (1.01)	5.96 (0.95)	5.77 (1.09)	5.36 (1.08)
Backward digit recall_span	5.33 (1.24)	5.13 (1.42)	4.94 (1.21)	4.65 (1.41)	4.60 (1.00)	4.00 (1.46)	3.64 (1.35)
TMT_A_time	29.71 (12.40)	28.07 (11.56)	32.58 (13.52)	32.11 (15.18)	38.18 (12.70)	49.42 (22.15)	70.70 (31.97)
TMT_B/A ratio	0.53 (0.28)	0.54 (0.28)	0.55 (0.28)	0.52 (0.25)	0.49 (0.22)	0.63 (0.35)	0.45 (0.23)

Data are presented as means with standard deviations in parentheses for each age group.

## Data Availability

The datasets generated during and/or analyzed during the current study are not publicly available for privacy or ethical restrictions; however, they will be available from the corresponding author upon reasonable request.

## Supplementary Materials

The following supporting information can be downloaded at: https://www.mdpi.com/article/10.3390/brainsci15101120/s1, File S1: A description of syntactically complex structures. References [76,77,88,89,90,91,92] are cited in the supplementary materials.

## Abbreviations

The following abbreviations are used in this manuscript:

AAT	Aachen Aphasia Test
CRIq	Cognitive Reserve Index Questionnaire
LLL	Lifelong Learning
MoCA	Montreal Cognitive Assessment
TMT	Trail Making Test
