# Supplementary material for: Aging, Cognitive Efficiency, and Lifelong Learning: Impacts on Simple and Complex Sentence Production During Storytelling
Source: Brain Sci. 2025 Oct 18;15(10):1120. doi: 10.3390/brainsci15101120 (PMC12564211; doi:10.3390/brainsci15101120)
Supplement: Supplementary file 1 [file brainsci-15-01120-s001.zip › brainsci-3880534-supplementary.pdf]

## Supplementary file

*A description of syntactically complex structures*

Depending on their syntactic structure, sentences can be categorized as simple or complex. Simple sentences (1) consist of a single independent clause made up of obligatory clausal elements such as verb, subject, and object, and optional clausal elements such as adverbials, and complements [88].

(1) The man climbed imprudently the tree in the garden.

Obligatory elements in (1) are the subject (*the man*), the verb (*climbed*), and the direct object (*the tree*), while optional elements are the adverb (*imprudently*), and the complement (*in the garden*).

Complex sentences present two or more clauses linked together through coordination or subordination. Coordination implies a balance between two or more independent clauses that present similar semantic value in the sentence. The sentence in (2) show an example of coordination, where two clauses are linked together by the coordinating conjunction *and*. Since the two clauses present similar semantic value, they can be produced separately (e.g. *The man climbed the tree*; *The tree is green*).

(2) The man climbed the tree and the tree is in the garden.

Subordination implies that one or more clauses hold less semantic value than the main clause they are linked to. The sentence in (3) shows an example of subordination, where two clauses are linked together by the complementizer *that*. Conversely, the subordinate clause (*that is in the garden*) cannot be produced alone since it is ungrammatical.

(3) The man climbed the tree that is in the garden.

Subordinate clauses can be classified in complement clauses (4), adverbial clauses (5), and relative clauses (6a-b).

(4) The mother did not notice that the boy has climbed on the stool.
(5) The man broke a leg because he fell from the tree.
(6) a. The woman that is blond calls the people that live in the house.
b. The woman calls the people that live in a house nearby.

Complement clauses serve as one of the arguments of the verb in the main clause in order to complete verb argument structure [89]. Therefore, they can serve as the subject, the direct object or the indirect object of the main verb. For example, in (4) the subordinate clause (*That the boy has climbed on a stool*) is the object complement of the main clause. Adverbial clauses are not required by the verb and serve as adjuncts to the main clause. They are linked based on semantic features mainly resorting to temporal (e.g. "*when*") or causal (e.g. "*because*") connectives. For example, the subordinate clause in (5) is introduced by the conjunction *because* and it is not necessary to complete the main sentence (*The man broke a leg*). Relative clauses are subordinate clauses that modify a nominal element (i.e. the noun phrase, NP) in the main clause [90]. They can be appositive

if they add additional non-essential information about the nominal element they refer to as in (6a), or they can be restrictive if they modify the nominal element limiting the number of its referents as in (6b). Relative clauses are considered particularly difficult since they are derived by syntactic movement. Syntactic movement refers to the detachment of a sentence constituent from the position where it is generated and interpreted as a verb argument to a new position, typically at the beginning of the sentence, where it is produced. When the constituent moves, it leaves a trace or copy in its base position. The copy/trace receives the thematic role from the verb, filling its argumental structure, and spread it with the moved element, with which it is coindexed [91].

Depending on the position where the moved element is interpreted, it is possible to define the type of relative clause. Subject relative clauses (7a) are derived by the movement of the subject, object relative clauses (7b) are derived from the movement of the direct object, and oblique relative clauses (7c) are derived by the movement of an indirect object.

(7) a. The woman looks at the man that ___ climbed the tree.
b. The woman looks at the tree that the man climbed ___.
c. The dog looks at the man to whom the woman gives the coffee ___.

As shown in examples (7a) and (7b), subject and object relative clauses are usually introduced by the complementizer "*that*", while oblique relative clauses (7c) present a more complex structure and are introduced by a preposition and a relative pronoun.

Passive sentences can be considered as complex structures even though their structure does not imply the presence of more than a clause. They are characterized by a reorganization of the grammatical functions found in the active sentences as showed in (8), namely the direct object (*the dishes*) of the active sentence becomes the subject in the passive sentence, and the subject of the active sentence (*the mother*) can be optionally expressed as a prepositional phrase introduced with the preposition *by*.

(8) a. The woman washes the dishes.
b. The dishes are washed by the woman.

These sentences are acquired later and in several steps by children (for a review: Guasti, [92]), and their processing was found difficult for several adult populations such as university students with dyslexia [75], and persons with aphasia [76,77].

Passive sentences as well object relative clauses, and all the sentences derived by the movement of the object, are particularly demanding because of the violation of the canonical order of the thematic roles. Indeed, in simple sentences the Agent (e.g., the entity that begins the action) precedes the Theme or Patient (e.g., the entity that undergoes the action performed by the Agent), while in complex sentences, such as passives and object relatives, the Theme or Patient of the sentence precedes the Agent causing, especially in persons with language and cognitive impairments, a misprocessing of the sentence.
